# Child Witness Expressions of Certainty Are Informative

**DOI:** 10.1037/xge0001049

**Published:** 2021-09-09

**Authors:** Alice A. Winsor, Heather D. Flowe, Travis M. Seale-Carlisle, Isabella M. Killeen, Danielle Hett, Theo Jores, Madeleine Ingham, Byron P. Lee, Laura M. Stevens, Melissa F. Colloff

**Affiliations:** 1 School of Psychology, University of Birmingham; 2 Wilson Centre for Science and Justice, Duke University; 3 Department of Psychology, University of California, San Diego

**Keywords:** confidence and accuracy, development, eyewitness identification, metacognition, signal-detection

## Abstract

Children are frequently witnesses of crime. In the witness literature and legal systems, children are often deemed to have unreliable memories. Yet, in the basic developmental literature, young children can monitor their memory. To address these contradictory conclusions, we reanalyzed the confidence–accuracy relationship in basic and applied research. Confidence provided considerable information about memory accuracy, from at least age 8, but possibly younger. We also conducted an experiment where children in young (4–6 years), middle (7–9 years), and late (10–17 years) childhood (*N* = 2,205) watched a person in a video and then identified that person from a police lineup. Children provided a confidence rating (an explicit judgment) and used an interactive lineup—in which the lineup faces can be rotated—and we analyzed children’s viewing behavior (an implicit measure of metacognition). A strong confidence–accuracy relationship was observed from age 10 and an emerging relationship from age 7. A constant likelihood ratio signal-detection model can be used to understand these findings. Moreover, in all ages, interactive viewing behavior differed in children who made correct versus incorrect suspect identifications. Our research reconciles the apparent divide between applied and basic research findings and suggests that the fundamental architecture of metacognition that has previously been evidenced in basic list-learning paradigms also underlies performance on complex applied tasks. Contrary to what is believed by legal practitioners, but similar to what has been found in the basic literature, identifications made by children can be reliable when appropriate metacognitive measures are used to estimate accuracy.

Each year, millions of children around the world become witnesses or victims of crime. In 2018 in England and Wales, one in ten children aged 10–15—that is, 841,000 children—were victims (Office for National Statistics, 2018). In the United States, more than one million children were victims (Bureau of Justice Statistics, 2018). Although the prevalence of child victimization does not seem to fluctuate greatly over time (e.g., Office for National Statistics, 2017, 2018), children’s testimonies are becoming increasingly present in Criminal Justice Systems worldwide. In the United Kingdom, for example, the number of children contributing memory evidence by providing police statements and courtroom testimony increased by 60% over a four-year period (2006–2009; Plotnikoff & Woolfson, 2011). Children as young as 2 years old provide memory evidence (e.g., Bowcott, 2017; *R v. Barker,* 2010). Despite the increasing reliance on memory evidence from children, surprisingly little research has investigated the reliability of children’s memory reports. Moreover, the existing applied eyewitness literature compared with the basic developmental and metacognitive literatures yield conflicting conclusions. To better understand the divide between basic and applied research, we review the literature and reanalyze data from both fields. To bridge the divide, we also conduct a new experiment testing children aged between 4 and 17 to examine the reliability of one type of memory evidence—the identification of a previously seen culprit from a police identification parade.

## Identification Parades and Memory Accuracy

When the identity of the culprit is unknown, a child witness may be asked to make an identification from a police identification parade (hereafter, a lineup). There are no official statistics on the number of children aged under 18 who view lineups each year, but given the proportion of children who experience crime there is reason to believe that the number is substantial. Recently, we surveyed 48 police officers from a U.K. metropolitan police force, and they estimated, on average, that 18% of child witnesses attempt to make an identification from a lineup. During a police lineup, the witness is shown images of the police suspect and other individuals who look similar to the suspect and are known to be innocent, called *fillers*. The police suspect may be innocent or may be guilty (i.e., may or may not be the culprit). It is the job of the witness to identify the culprit if they are present in the lineup or reject the lineup if the culprit is not present.

To determine the likely accuracy of children’s lineup identification decisions, applied research has largely focused on measuring average *memory discrimination accuracy*—that is, ability to discriminate between innocent and guilty suspects—in children of different age groups. This research suggests that memory discrimination accuracy improves with age (Humphries & Flowe, 2015; Laurence & Mondloch, 2016; see Fitzgerald & Price, 2015 for a meta-analysis). There is some discussion about the mechanisms underlying the improvements in memory discrimination accuracy with age on lineup tasks. There has been a long tradition in the eyewitness literature of research concluding that children aged from about 5 years are just as likely as their older peers (and even adults) to make a correct identification of a guilty suspect in a target-present lineup, and that age differences in lineup identifications are limited to older children making fewer mistaken identifications of innocent suspects from target-absent lineups, perhaps due to younger children having difficulty withholding an inappropriate response (e.g., Dunlevy & Cherryman, 2013; Havard & Memon, 2013; Pozzulo & Lindsay, 1998; see also Roebers & Spiess, 2016; Schneider & Löffler, 2016; for discussion on maturation of monitoring and cognitive control in the basic science literature). Yet, some eyewitness research with children has found that correct identifications of guilty suspects in target-present lineups increase with age, possibly because memory mechanisms gradually mature throughout childhood (for example, Brewer & Day, 2005; Fitzgerald et al., 2014; Fitzgerald & Price, 2015; Keast et al., 2007; see also Crookes & McKone, 2009; McKone et al., 2012; for debate on development of face identification abilities in the basic science literature). Despite ongoing discussion about the underlying mechanisms, research has concluded that average *memory discrimination accuracy* improves throughout childhood.

Applied research with adult witnesses, however, indicates that average memory accuracy is not the most important metric for determining the reliability of eyewitness identifications. A better metric for legal decision-makers to decide how much trust to place in witness memory evidence is to use metacognitive measures, such as confidence judgments (e.g., Mickes, 2015). This is because, regardless of their average memory discrimination accuracy, a person with a reliable memory has good metacognitive ability^1^ and is able to appropriately modulate their confidence in response to their memory performance, reporting higher confidence when likely to be correct and lower confidence when not likely to be correct (Fleming & Lau, 2014). Even if memory discrimination accuracy is relatively poor, the reliability of memory evidence can be good, because people can be aware when their memories are inaccurate or accurate (e.g., Brewer & Wells, 2006; Sauer et al., 2010).

A key question is whether children can monitor their memory accuracy. Answering this question is practically important in determining how child witness memory evidence should be interpreted in legal systems and theoretically important in developing a unified theory of children’s metacognitive development. Currently, contradictory conclusions have been drawn in the applied witness and basic developmental literatures.

## Memory Monitoring in Children

In the witness identification literature, the consensus is that children are unreliable witnesses, because their confidence judgments do not reflect their memory accuracy (Keast et al., 2007; Powell et al., 2013). For example, one influential study asked children (aged 10–14) to watch a mock-crime video and later identify the culprit and another individual in the video (Keast et al., 2007). The researchers found that the correspondence (called *calibration*) between confidence and accuracy was poor and concluded that a child’s confidence provides no useful indicator of a suspect’s innocence or guilt. Similar conclusions have been reached in other research recruiting children who are between the ages of 8 and 11 (Brewer & Day, 2005; Parker & Carranza, 1989; Parker & Ryan, 1993). Thus, the witness literature suggests that children who are younger than 12 have not yet fully developed the skills to monitor their memory, or to use confidence scales to indicate accuracy (Powell et al., 2013; but see Bruer et al., 2017 for a notable exception). Critically, this conclusion has informed legal guidance worldwide. For example, Powell et al. (2013) state that confidence is not a useful guide to accuracy for children’s identification responses, and this book has been cited by superior courts in every jurisdiction in Australia and New Zealand.

Yet, a more positive picture emerges when the developmental literature is considered. Developmental research suggests that children from about age 4 or 5 can demonstrate memory-monitoring skills, which improve throughout childhood (Sodian et al., 2012). For example, in one study that is representative of the basic literature, children aged 3–5 viewed objects and then subsequently identified which object of two they had seen before and provided a confidence judgment after each decision. Children from age 4 provided higher confidence judgments, on average, for correct answers than incorrect answers (Hembacher & Ghetti, 2014), thereby demonstrating memory-monitoring skills. Moreover, instead of collecting confidence judgments (an *explicit* metacognitive judgment), other researchers have found that young children from age 3 can appropriately express uncertainty *implicitly* without full awareness, using gestures like shaking their head, shrugging their shoulders (Kim et al., 2016), or asking for help when they are unsure (Ghetti et al., 2013; Goupil et al., 2016). Although the memory task and test format can moderate the accuracy of children’s memory monitoring (e.g., Steiner et al., 2020), taken together, the developmental literature suggests that children from age 3 can monitor their performance when implicit measures of metacognition are collected, and that children from age 4 or 5 have developed at least some memory-monitoring skills and the ability to use explicit confidence scales to indicate accuracy.

Why has basic developmental research generally concluded that children’s expressions of certainty can be informative about memory accuracy, whereas applied witness research has concluded the opposite? There are at least three possible reasons. The first reason might be the task itself: Memories from complex witnessed events (e.g., the physical appearance of a culprit) may be more difficult for younger children to monitor, compared with the simple to-be-remembered stimuli (e.g., pictures) that children monitor in the developmental literature (Harris, 1995). A second reason might be that different methods have been used to measure memory-monitoring across the literatures. For example, eyewitness researchers have seldom measured implicit metacognition, such as a child’s behavior during the lineup task, which might be more predictive of accuracy in younger children than explicit confidence judgments (e.g., Ghetti et al., 2013; Kim et al., 2016). A third reason is differences in statistical approach in analyzing explicit confidence judgments. A common approach in the developmental literature is to calculate average confidence for correct versus incorrect decisions, but this does not provide all of the information relevant to examine memory monitoring, because there could be a poor correspondence between confidence and accuracy, even if confidence is, on average, higher for correct than incorrect decisions. A good correspondence between confidence and accuracy occurs when high-confidence decisions are highly accurate, medium-confidence decisions are moderately accurate, and low-confidence decisions are of low accuracy. Conversely, because legal decision-makers (e.g., judges, jurors) are interested in determining the likelihood of accuracy of a single identification made with a particular level of certainty, eyewitness researchers have measured the typical correspondence between witnesses’ certainty judgments and their average accuracy. Examining the correspondence between certainty and accuracy provides comprehensive information about memory monitoring skills, but the applied literature has used approaches that can underestimate the relationship between confidence and accuracy. We explain this in more detail next.

## Measuring the Relationship Between Confidence and Memory Accuracy

The witness identification literature has traditionally relied on statistical techniques which can underestimate the confidence–accuracy relationship. For example, the point biserial correlation coefficient has been used, but we now know that the correlation coefficient can vary dramatically, even when confidence and accuracy are perfectly calibrated, because it is affected by the distribution of correct and incorrect identification decisions across confidence levels (Juslin et al., 1996). Compared with the point biserial correlation coefficient, a better way to assess the relationship between confidence and accuracy is to plot subjective confidence against objective performance (proportion correct) to construct calibration curves and calculate associated calibration statistics (e.g., Over/under confidence, C, Adjusted Normalized Resolution Index). More recent research has used the calibration approach to advance understanding about metacognition (e.g., Keast et al., 2007; Palmer et al., 2013; Sauer et al., 2010). From an applied perspective, however, calibration analyses may also underestimate the informativeness of confidence in criminal justice settings, because it includes filler IDs along with innocent suspect IDs to calculate errors (Mickes, 2015; Wixted & Wells, 2017). When legal decision-makers are determining the likely accuracy of a witness’s identification, they are determining the likely accuracy of an identification of a police suspect. This is because only suspect identifications (and not filler identifications) are used as evidence of a suspect’s guilt or innocence in court (Wixted & Wells, 2017). Consequently, instead of calibration analyses, researchers have recently begun to use confidence accuracy characteristic (CAC) analysis to examine the reliability of witness identification decisions.

In a CAC analysis, subjective confidence is plotted against objective performance, but only innocent suspect IDs (and not fillers) are included when calculating errors (Mickes, 2015). Recent research in the adult witness literature using CAC analysis suggests that there is generally a strong relationship between confidence and suspect ID accuracy in adults (e.g., see Wixted & Wells, 2017, for a review). Confidence typically tracks suspect ID accuracy, even in situations where overall memory discrimination accuracy is comparatively poor, such as in older adults compared with younger adults (Colloff et al., 2017), or in those who experienced a longer delay between encoding and the identification test (Wixted et al., 2016). To explain why confidence typically tracks suspect ID accuracy, even in situations where overall memory discrimination accuracy is comparatively poor, we need to consider theoretical models from basic science. In this regard, a constant likelihood ratio signal-detection model from the broader memory literature has recently been applied to account for adult witness memory performance (Colloff et al., 2017; Semmler et al., 2018; Stretch & Wixted, 1998).

## Constant Likelihood Ratio Signal-Detection Model

The constant likelihood ratio signal-detection model posits that adults “fan out” their confidence criteria across a memory strength continuum in conditions yielding poorer memory discriminability. The idea is that when discrimination accuracy is lower, adults place their most conservative decision criterion (e.g., 100% confidence) at a more conservative location on the memory strength continuum (requiring more memory evidence to make a recognition memory decision with high confidence), while placing their liberal decision criterion (e.g., 10% confidence) at a more liberal location (requiring less memory evidence to make a decision with low confidence). Behaving in this way means that adults place their decision criteria optimally to maintain a constant likelihood of accuracy at each level of confidence over hard (poorer discrimination) and easy (better discrimination) conditions.^2^ It has been proposed that adults learn how to place their confidence criteria optimally through a lifetime of error feedback training about the circumstances in which their memories are and are not accurate (Mickes et al., 2011; Stretch & Wixted, 1998). The constant likelihood ratio signal-detection model has been applied to account for performance of older adults, showing that they optimally place their criteria to compensate for age-related decline in memory performance (Colloff et al., 2017) and also to show that adults optimally place their criteria to compensate for viewing distance impairments on memory performance (Semmler et al., 2018). As such, theory predicts and data suggest that, at least as adults, eyewitnesses can be reliable; they have metacognitive skills to monitor memory and can usually assign appropriate confidence judgments that reflect their identification accuracy. We considered whether and at what age children optimally place their decision criterion and assign appropriate confidence judgments that correspond to their memory accuracy.

## The Current Study

Currently, it is unclear why the basic and applied literatures have reached different conclusions regarding the informativeness of children’s expressions of certainty. It is important to reexamine memory-monitoring in children for both basic and applied researchers. First, for basic researchers, theories should account for monitoring performance across task domains. If it is the case that children can monitor their memory on a complex eyewitness identification task, and show a strong correspondence between certainty and accuracy, this suggests that the fundamental architecture of metacognition that has previously been evidenced in the developmental literature on relatively simple tasks also underlies performance on complex tasks. Conversely, if children do not have a good metacognitive awareness on a complex task, this suggests that the ability to monitor accuracy is dependent on the cognitive activity, or complexity of the memory, being monitored (Ghetti et al., 2013). Second, for applied researchers, the correspondence between certainty and accuracy (i.e., the reliability of children’s identification decisions) may currently be underestimated in legal systems worldwide, because young children are able to monitor their memories according to studies in the basic developmental literature; and the most appropriate statistical techniques have not been used. Theoretically, a constant likelihood ratio signal-detection model predicts that people optimally adjust their criterion, and the correspondence between certainty and accuracy will improve with age, as the quantity of memory error-feedback training increases.

In this study, we first use CAC analysis to reanalyze children’s explicit confidence judgments in basic list-learning memory studies and an influential eyewitness identification study that sampled children in late childhood (Keast et al., 2007). The data (both basic and applied) show a strong relationship between confidence and accuracy in children. We then present an original eyewitness study in which we asked more than 2,220 children in young (aged 4–6), middle (aged 7–9), and late (aged 10–17) childhood to watch a video of a complex event, then attempt to identify the person who was in the video from a police lineup, and provide a confidence judgment (explicit measure of metacognition). We used a novel interactive lineup—in which the lineup faces can be rotated and viewed from different angles—to record children’s viewing behavior moment by moment and explore whether viewing behavior (implicit measure of metacognition) differs in children who made correct versus incorrect identifications. Again, contrary to what is believed to be true in legal systems around the world, but consistent with the basic literature, we show that children’s expressions of certainty are informative even on a complex memory task.

## Reanalysis of Children’s Explicit Confidence Judgments

To date, the basic developmental and applied eyewitness literatures have coexisted, with little communication, yielding conflicting conclusions. Missing from the literature is a comprehensive overview of the confidence–accuracy relationship in children. What do the data typically look like—in both the basic and applied literatures—when accuracy is plotted as a function of confidence?

### Developmental Research

A few basic recognition memory studies in children have collected explicit confidence ratings. These studies typically report mean confidence for correct responses versus mean confidence for incorrect responses to measure children’s proficiency in *uncertainty monitoring*. The existence of uncertainty monitoring in children suggests that confidence can be informative with respect to accuracy. If children express significantly higher confidence for their correct answers compared with their incorrect answers, it is concluded that they are able to monitor their own uncertainty. Nevertheless, studies of uncertainty monitoring generally do not directly characterize the confidence–accuracy relationship in children. For example, even if a child is able to differentiate when they are correct from when they are incorrect using confidence, this does not speak to the correspondence between confidence and accuracy or the absolute accuracy of their memory at different levels of confidence (e.g., it does not mean that high-confidence accuracy is very high, or low-confidence accuracy is very low). Measures of uncertainty monitoring and the confidence–accuracy relationship (i.e., CAC analysis) are likely to have some level of redundancy. More likely than not, children who show uncertainty monitoring will also show a clear confidence–accuracy relationship. But how accurate, exactly, is a decision made with high confidence?

For basic memory studies, CAC analysis is equivalent to proportion correct (or positive predictive value), plotted as a function of confidence. In studies in which relevant data were shown only in a plot, near exact values were estimated using WebPlotDigitizer (https://automeris.io/WebPlotDigitizer/; Wixted & Wells, 2017). As will become clear, when data from the developmental literature are plotted using CAC analysis, a strong relationship between confidence and accuracy in children exists across a range of stimuli and experimental tasks.

#### Berch and Evans (1973)

**Figure 1 fig1:**
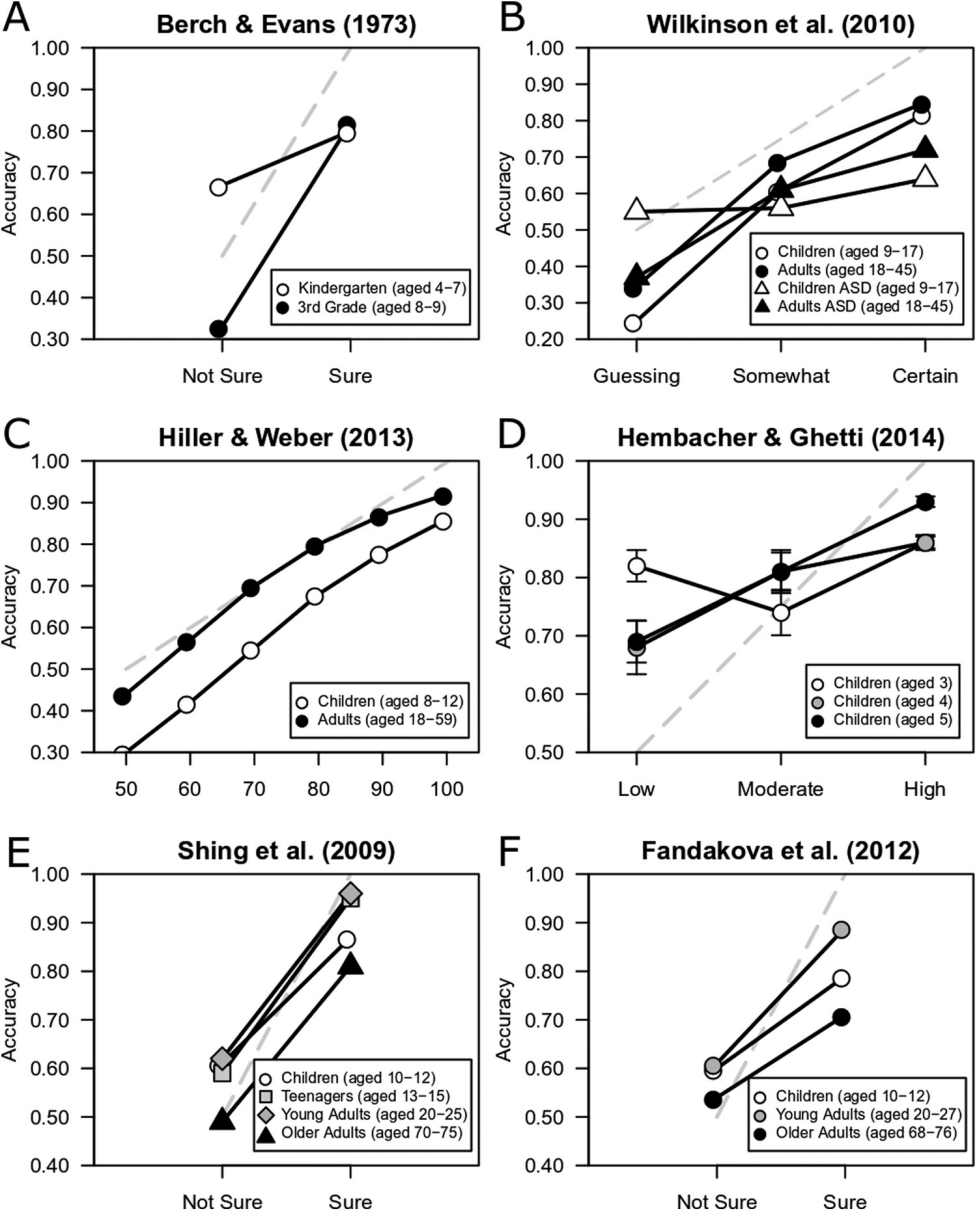
A Confidence Accuracy Characteristic Reanalysis of Data From Six Basic List-Learning Memory Experiments, Plotting Accuracy (Proportion Correct) as a Function of Confidence *Note.* On each plot, the dashed line indicates chance-level performance at the lowest confidence bin and perfect performance at the highest confidence bin. In D, error bars are ±1 *SE.*

Berch and Evans (1973) tested 4- to 9-year-old children on a continuous recognition task. Children were shown a set of 90 cards with two-digit numbers on them. There were 45 unique numbers, so each number appeared twice in the set. The set was divided into three blocks of 30 cards, with 15 “new” items and 15 “old” items appearing in each block. New items were numbers that had not been seen before in that block and old items were numbers that had been seen once previously. The children were asked to state whether each number shown was new or old and to rate their confidence in each of their decisions (*sure* vs. *not sure*). Berch and Evans (1973) analyzed their results using a probability function for old/new judgments as a function of confidence. We estimated the posterior probability values for old decisions using WebPlotDigitizer, because an old decision is analogous to making a positive identification in an eyewitness identification paradigm. Figure 1A shows these data plotted. For third graders (aged 8–9), the confidence–accuracy relationship was strong, because as confidence increased so did accuracy. Accuracy was 33% correct for low-confidence responses and 82% correct for high-confidence responses. The confidence–accuracy relationship was less strong for kindergarteners (aged 4–7): Accuracy was 67% correct at low confidence and 80% correct at high confidence. The dashed line in Figure 1A illustrates chance performance at the lowest confidence rating, and perfect performance at the highest confidence rating. It is apparent that children were slightly overconfident, because ∼80% correct at the highest level of confidence is lower than perfect performance (i.e., 100% correct). Nevertheless, it is clear that children’s confidence ratings were informative about likely accuracy.[Fig fig1]

#### Wilkinson et al. (2010)

Wilkinson et al. (2010) compared typically developing children between 9 and 17 years old with children with Autism Spectrum Disorder (ASD) of the same age using an old/new face recognition paradigm. They also compared adults (18 to 45 years) with and without ASD. During the learning phase, participants viewed 24 female faces sequentially. During the testing phase, participants were shown 48 faces, 24 old (i.e., shown in the learning phase) and 24 new (i.e., not shown in the learning phase). The faces were presented one at a time and participants decided if each face was old or new and rated their confidence (*guessing*, *somewhat certain*, or *certain* for adults; and *guessing*, *somewhat sure*, or *sure* for children). We plotted proportion correct as a function of accuracy. Figure 1B illustrates that the typically developing children showed a strong confidence–accuracy relationship, while the children with ASD showed no relationship. Typically developing children were 25%, 60%, and 82% accurate at low, medium, and high confidence. Of note is that the CAC for the typically developing children mirrors that of the typically developed adults, although children were less accurate at low and medium confidence. In both typically developing children and adults, however, high-confidence decisions were likely to be accurate (85% and 82% correct in adults and children, respectively).

#### Hiller and Weber (2013)

Hiller and Weber (2013) tested 8- to 12-year-old children and adults (18–59 years) using an associative word-pair recognition paradigm. Participants were shown 28 word-pairs sequentially in the encoding phase and, after a two-minute delay, were given a memory test also consisting of 28 word-pairs. Participants had to recognize each word pair in the test as old or new, and after each decision rate their confidence using a confidence scale that ranged from 50 (*guessing*) to 100 (*certain*). Hiller and Weber plotted predicted log odds of a recognition decision being correct or incorrect as a function of confidence. We converted the predicted log odds to proportion correct and again focused on old decisions. Unsurprisingly, Figure 1C indicates that adults showed a strong confidence–accuracy relationship and were more accurate at each level of confidence than the children. Children’s accuracy was approximately 30% correct for low-confidence decisions, and accuracy increased monotonically with confidence, up to 86% correct for high-confidence decisions. Thus, the confidence–accuracy relationship was strong for both adults and children.

#### Hembacher and Ghetti (2014)

Hembacher and Ghetti (2014) tested uncertainty monitoring in 3- to 5-year-old children using a two-alternative forced-choice object recognition task. During the learning phase the children viewed 30 drawings of common objects. During the test phase, children decided which of two drawings they had seen in the learning phase and made a confidence judgment on a 3-point picture scale. Each point on the confidence scale was an illustration of a child displaying a facial and body expression, indicating either low, moderate, or high confidence. We obtained the data for this study through the Open Science Framework and plotted proportion correct as a function of confidence. Figure 1D illustrates that 3-year-olds show virtually no confidence–accuracy relationship, with both low-confidence and high-confidence responses resulting in similar levels of overall accuracy (both around 84%). Four-year-olds show a moderate confidence–accuracy relationship, with low-confidence responses being 68% correct and high-confidence responses being 86% correct. Five-year-olds showed a strong confidence–accuracy relationship with low-confidence responses being 69% correct and high-confidence responses being approximately 93% correct. These findings echo Hembacher and Ghetti’s conclusions about the developmental trajectory of uncertainty monitoring in their original analysis, whereby 3-year-olds were unable to monitor their own uncertainty, 5-year-olds were able to monitor their uncertainty, and 4-year-olds fell somewhere in between.

#### Shing et al. (2009)

Shing et al. (2009) tested children (aged 10–12), teenagers (aged 13–15), young adults (aged 20–25), and older adults (aged 70–75), using a word-pair associative recognition task, to examine age differences in high-confidence errors. A word in the participant’s native language was paired either with a second native language word (for the control group) or a foreign language word (for the experimental group). We focused on the performance of the control group. Participants were shown a list of 45 word-pairs and then shown 60 pairs and asked to decide whether pairs were old (i.e., as seen at study) or new (i.e., words rearranged into previously unseen pairs), rating their confidence on a 3-point scale (ranging from 1 *unsure* to 3 *sure*). Shing et al. also manipulated whether participants at encoding were informed of a memory strategy (poststrategy), or not (prestrategy). Shing et al. reported the percent *sure* responses as a function of the hit and false alarm rates for each condition on a plot, but not the percent of responses that were made with a confidence rating of 1 (*unsure*) or 2. The percent *sure* responses represents the proportion of the participants’ responses that were made with high confidence. We multiplied the percent *sure* hits by the overall hit rate, thereby estimating the high-confidence hit rate, and also multiplied the percent *sure* false alarms by the overall false alarm rate, estimating the high-confidence false alarm rate. Because the data for confidence ratings 1 and 2 were not reported separately, we assumed all responses not rated *sure* were considered *not sure* or low confidence. The proportion of *not sure* hits was the hit rate minus the *sure* hit rate. Similarly, the proportion of *not sure* false alarms was the false alarm rate minus the *sure* false alarm rate.

Proportion correct for each age group, averaged across the pre- and poststrategy conditions, is displayed in Figure 1E. Children showed a strong confidence–accuracy relationship, although not quite as strong as teenagers and young adults. For low-confidence responses children were approximately 61% accurate, and for high-confidence responses children were approximately 87% accurate. Teenagers and young adults achieved approximately 95% and 96% accuracy at high confidence. Older adults also showed a confidence–accuracy relationship, but they were more overconfident at high confidence than children. On average, for high-confidence responses older adults were only 81% accurate. The task was designed to be particularly difficult for older adults, because older adults show deficits in associative binding; however, it is worth highlighting that children, on average, achieved higher accuracy than older adults, at both low and high confidence.

#### Fandakova et al. (2013)

Fandakova et al. (2013) tested children (aged 10–12), young adults (aged 20–27), and older adults (ages 68–76), using a repeated continuous recognition task of word pairs, to examine age differences in high-confidence errors, across three consecutive blocks of the experiment. In each block, participants saw word pairs that they had never seen before (novel), word pairs that had not been seen in that particular block but had been seen previously in a block (lure pairs), and word pairs that had been seen in that block but had been rearranged (rearranged pairs). Participants had to decide whether a word-pair was old (i.e., exactly the same as a word-pair that they had seen in that block) and responded on a 4-point scale (*sure new*, *unsure new*, *unsure old*, *sure old*). Fandakova et al. (2013) plotted the overall proportion of hits and false alarms (to lure pairs and rearranged pairs) for each block, as well as the proportion *sure* hits and proportion *sure* false alarms. To calculate the proportion of *not sure* hits and false alarms, we used the same approach that we took for Shing et al. and assumed all responses not considered *sure* were considered *not sure* or low-confidence and thus subtracted the proportion *sure* hits and false alarms from the overall proportion of hits and false alarms. We averaged over the two types of lures (lure pairs and rearranged pairs) to calculate the false alarm rate, and we also averaged over the hit and false alarm rates across the three blocks.

Proportion correct as a function of confidence for each age group is shown in Figure 1F. As in Shing et al. (2009), children showed a strong confidence–accuracy relationship, although not as strong as young adults. Children were 60% correct for low-confidence responses and 79% correct for high-confidence responses, and young adults were 61% and 89% correct for low- and high-confidence responses, respectively. Again, as in Shing et al. (2009), older-adults were more overconfident at high confidence than children. On average, for high-confidence responses older adults were only 71% accurate. Again, it is not surprising that this associative recognition task was particularly difficult for older adults. However, it is worth noting that, even in young adults, highly confident decisions were not as close to perfect accuracy (i.e., 100% correct) as one might expect. It seems that there was something about this task that caused the relationship between confidence and accuracy to be weaker than usual. Nevertheless, it is still the case that in all age groups—including children—there was a relationship between confidence and accuracy because high-confidence responses were more accurate than low-confidence responses.

In sum, a reanalysis of data from basic list-learning paradigms shows that across a variety of memory tasks and ratings scales, a relationship between confidence and accuracy exists in children from at least aged 8 (Berch & Evans, 1973; Fandakova et al., 2013; Hiller & Weber, 2013; Shing et al., 2009; Wilkinson et al., 2010), and perhaps even from around age 5 (Hembacher & Ghetti, 2014). Although the confidence–accuracy relationship in children is not as strong as it is in adults, and children were slightly overconfident at high-confidence, it is nevertheless clear that children are still reasonably accurate (∼85% correct) when expressing high confidence and are less accurate (closer to chance performance) when they express low confidence. This indicates that children are generally aware about the strength of a memory signal and are using it to make a decision. The next question is whether the confidence–accuracy relationship is also strong for children on an eyewitness identification task.

### Eyewitness Research

Despite a large literature on the calibration between confidence–accuracy in adult witnesses, to our knowledge only two notable studies have used calibration analysis with children in a witness identification paradigm (Brewer & Day, 2005; Keast et al., 2007). Both articles concluded that there were limitations in children’s memory monitoring processes, such that children’s confidence judgments were not informative about likely accuracy. Critically, these conclusions have informed legal guidance worldwide (e.g., Powell et al., 2013), and applied research on the topic has not been revisited. Only one of those two studies included both target-present and target-absent lineup conditions (Keast et al., 2007), and therefore we reanalyzed the data from Keast et al. (2007) using CAC analysis.

Keast et al. (2007) conducted two experiments with children in late childhood. In Experiment 1, children (*n* = 619, aged 10–13 years, *M* = 11 years 10 months) and adults (*n* = 600) viewed a simulated crime and attempted two separate identifications (of a thief and waiter) from eight-person lineups in which the target was either present or absent. Participants rated their confidence in their identification using a 0% to 100% confidence scale. The instructions provided to participants before the lineup were also manipulated to be either unbiased or biased, but this manipulation had little effect and therefore Keast et al. plotted calibration curves collapsed over both instruction conditions. In Experiment 2, children (*N* = 796, aged 10–14 years, *M* = 11 years 11 months) saw the same simulated crime and lineups as Experiment 1; but, before rating their confidence, half of the children received hypothesis disconfirmation (e.g., questions about conditions that could result in inaccurate eyewitness identifications), whereas the other half received control questions about their likes and dislikes. Keast et al. (2007) plotted calibration curves separately for each condition, so here we focus on the control condition.

**Figure 2 fig2:**
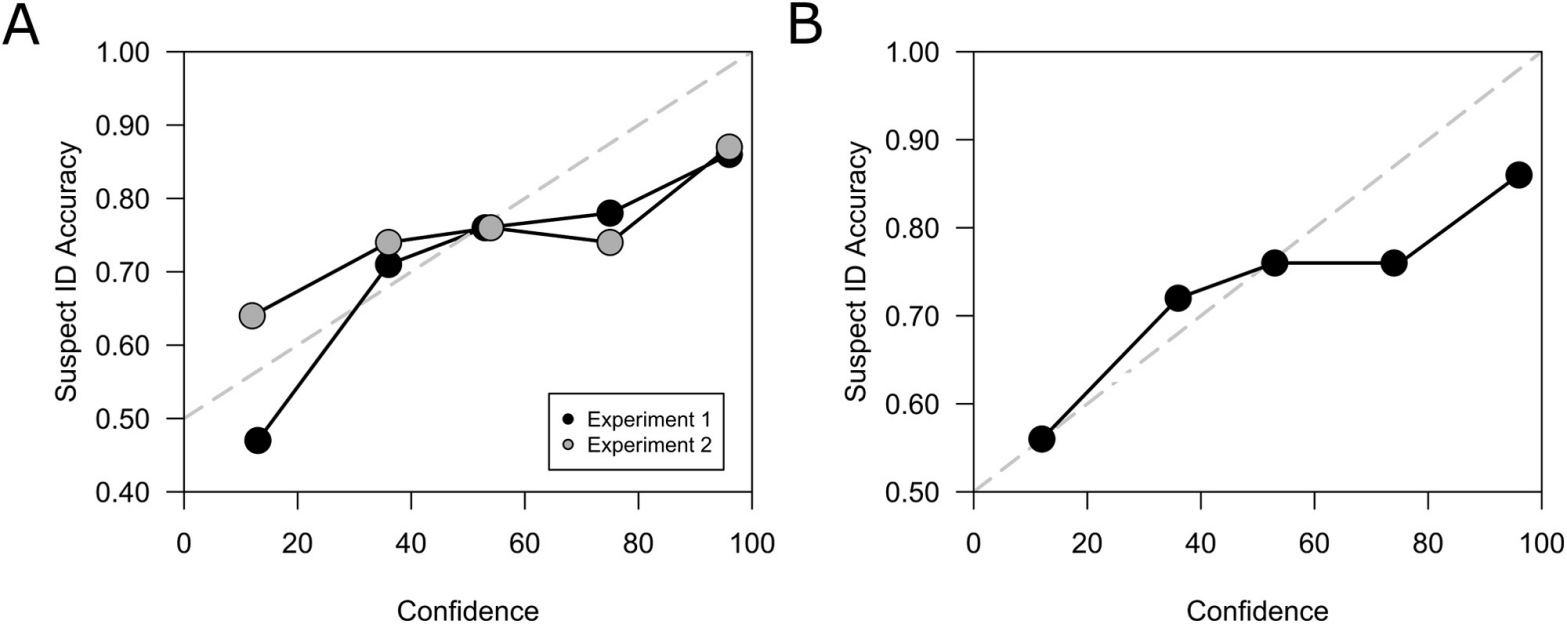
A Confidence Accuracy Characteristic Reanalysis of the Data From Keast et al. (2007); (A) Experiment 1 and 2 (Control Condition) and (B) Averaged Over Experiment 1 and 2 (Control Condition) *Note.* The dashed line indicates chance-level performance at the lowest confidence bin and perfect performance at the highest confidence bin.

To conduct CAC analysis, we first estimated the calibration data (i.e., percent correct at each level of confidence) using WebPlotDigitizer. As noted previously, the main difference between calibration and CAC analysis is the inclusion of the fillers in calibration analysis. Therefore, we next converted the aggregate accuracy scores into the Suspect ID scores required for CAC analysis by taking the reported accuracy score for a given level of confidence, *a1*, converting it to an odds score, *o*, where *o* = *a1*/(100 – *a1*), and then computing suspect ID accuracy, *a2*, using the formula *a2* = *o*/(*o* + 1/*n*), where *n* = lineup size (Wixted & Wells, 2017). Figure 2A shows the CACs for Experiment 1 (collapsed over both instruction conditions and over thief and waiter identifications) and Experiment 2 (data from the control condition, collapsed over thief and waiter identifications), and Figure 2B shows the average CAC collapsed over both experiments.[Fig fig2]

Figure 2 shows a relationship between confidence and accuracy in the Keast et al. (2007) child sample; as confidence increases, so does accuracy. Looking at the average CAC in Figure 2B, high-confidence suspect IDs (86% accurate) were more accurate than medium-confidence suspect IDs (76% accurate), which were more accurate than the low-confidence suspect IDs (56% accurate). Although children were overconfident at high confidence (i.e., were only 86% accurate when they were 100% confident, not 100% accurate when they were 100% confident), it is clear that confidence increased with accuracy. Consistent with the basic literature using list-learning paradigms, but in contrast to what the witness literature and legal systems believe to be true, this reanalysis indicates that a child’s expression of confidence provides considerable information about the likely accuracy of a suspect ID. Not only does the present reanalysis make that important point, it also reconciles what has previously seemed to be a contradiction between what has been learned about the confidence–accuracy relationship in children in the basic developmental literature and what has been learned about that relationship in the eyewitness identification literature. As it turns out, the contradiction seems to be more apparent than real. Whether tested using a basic list-memory paradigm or an eyewitness identification paradigm, a positive confidence–accuracy relationship in children *at least* 10 years old exits. Moreover, the basic literature has tested younger children and indicates a positive confidence–accuracy relationship in children from at least 8 years old, and perhaps even younger from age 5.

Next, we conducted an original eyewitness experiment with a broader age range of children than Keast et al. (2007) to examine how the confidence–accuracy relationship changed with age and examine whether implicit measures of metacognition were informative about accuracy in younger children, from age 4.

## Eyewitness Experiment

### Method

We preregistered our hypotheses and analysis plan before we collected data (https://osf.io/azs35), and our data are available (Winsor et al., 2018; https://osf.io/3zjd6/).

#### Design

We used a 3 (age: young, middle, late childhood) × 2 (lineup condition: target-present, target-absent) between-subjects design. Subjects were randomly allocated into one of the lineup conditions. Our data-collection stopping rule was to recruit at least 1,800 subjects and to continue data collection until we had at least 300 subjects in each of the between-subjects conditions. We planned to use receiver operating characteristic (ROC) analysis to measure memory discrimination accuracy, which requires large samples in lineup research, but the techniques for conducting power analyses are not well defined. ROC lineup studies typically recruit between 300 and 500 subjects per condition, and we followed this established convention. There were no previous child lineup ROC studies on which to base a basic power estimate but using the mean difference (mean difference = *p*AUC1 − *p*AUC2 = .052 − .037 = .015) and *SDs* (.07) observed in an adult eyewitness ID study comparing two lineup techniques (Mickes et al., 2012) as a guide, a subsequent power analysis for a one-tailed test indicated that, with 300 subjects per between-subjects condition, power for an ROC analysis would exceed 80%. This sample size was also large enough to plot stable CAC analysis curves. The research was reviewed according to the University of Birmingham Science, Technology, Engineering, and Mathematics Ethical Review Committee.

#### Subjects

Subjects were 2,228 children who we approached at a local children’s science museum and asked if they would like to take part in an experiment. Legal guardians provided informed consent for subjects aged under 16, and subjects aged 16 and 17 consented themselves. We excluded 23 children from the analyses because guardians interrupted or influenced the child’s answers. The final sample was therefore 2,205 children (aged: 4–17 years, *M* = 8.08 years, *SD* = 2.72 years; sex: 49% female; ethnicity: 81% Caucasian, 9% South Asian, 6% Mixed, 2% Black, 1% Chinese, 1% Other). Following our preregistered analysis plan, we divided the final sample into three age categories to achieve a relatively equal number of children in each category and to ensure at least 300 subjects in each cell of the (3 Age × 2 Lineup condition) design. In the young group there were 717 subjects, with a mean age of 5.16 (*SD* = .78, range 4–6). In the middle group, there were 848 subjects, with a mean age of 7.96 (*SD* = .81, range 7–9). Finally, in the late group, there were 640 subjects with a mean age of 11.50 (*SD* = 1.62, range 10–17).

#### Materials

##### Events

Ensuring variability in encoding and test conditions is important when trying to detect reliable and generalizable effects (Brewer et al., 2010; Lindsay et al., 1998). To this end, we filmed two videos that were appropriate to engage children. One video depicted a male in his late 20s tidying up children’s toys, and the other depicted a male in his mid-20s returning home with shopping and eating chocolate. Each video lasted approximately 70 s, showed the men’s faces from multiple angles, and had music playing in the background. Although these men did not commit a crime, we will refer to these males as culprits (or guilty suspects), as is common in the witness literature.

##### Lineups

The lineups were created to be interactive, using *Eyewitness Interactive Software* that we developed (Colloff et al., 2020). Subjects could use the laptop mouse to click on and rotate the lineup members 180° on the vertical axis to examine the faces from different angles. When the subject clicked on and rotated one lineup member, all of the lineup members rotated in unison, known as a simultaneous joint-movement lineup.

To create the lineups, we first captured each male culprit’s image. To select the fillers for each lineup, we created a modal description of each male culprit by asking a group of adult subjects (*N* = 20) to watch each video and, after each video, answer 10 multiple-choice questions about the male’s physical appearance (e.g., sex, race/ethnicity, age).^3^ We used the modal descriptions to select six other people who matched the description of each male culprit. For each culprit, the six selected faces constituted the target-absent lineup and we randomly selected one of the six faces to be substituted with the culprit to create the target-present lineup.

Police guidelines around the world state that police lineups should be fair; lineup members should be plausible alternatives to the suspect and the suspect should not stand out (e.g., Police and Criminal Evidence Act, 1984, Code D, 2011; Technical Working Group for Eyewitness Evidence, 1999). To ensure that our lineups were compliant with police guidelines, we conducted a mock-witness test with adults. Subjects from Amazon Mechanical Turk (*N* = 121, each remunerated .15 cents) read the modal description of each culprit, viewed a simultaneous joint-movement interactive lineup, and were asked to decide which person best fit the description. We calculated Tredoux’s *E*, a measure of *effective size*, which uses the distribution of mock-witness choices to determine how many members are appropriate (Tredoux, 1999). Tredoux’s *E* ranged from 4.27 to 4.80 (*M* = 4.51) across the four lineups (target-present and -absent lineups for each culprit). This result is consistent with assessments of effective size of lineups used as stimuli in experimental studies (e.g., Horry et al., 2015; Palmer et al., 2013) and real lineups created in police practice (e.g., Valentine & Heaton, 1999). This result indicates that our lineups were perceptually fair.

#### Procedure

Each child subject was tested individually, accompanied by a research assistant (RA) explaining the experimental task and inputting the subject’s responses on a laptop. Subjects were first asked their age, sex, and race/ethnicity; the answers of young children were confirmed with their guardian, who was close by. Subjects were told they were going to watch a video of a man named James and were asked to pay attention. Subjects put on headphones before the video began. Next, subjects watched a 2-min cartoon as a distraction task. Afterward, subjects removed their headphones, and we collected a preidentification confidence rating. Subjects were told:

In a moment, I am going to show you some pictures of different men. I want you to help me figure out if James (the man in the video) is one of the men in the pictures. There might be a picture of James in the group, or there might not be a picture of James in the group. Before I show you any pictures, I want you to tell me how sure you are that you would be able to correctly recognize James again, if you saw him in the group of photos.

Subjects were presented with a 5-point water-cup rating scale, ranging from *not at all sure* (empty cup) to *very sure* (full cup). The RA explained the water-cup rating scale following Bruer et al. (2017). In short, subjects were told that the amount of water in the cup reflected how sure they were, with more water meaning that they were more sure. Pilot testing confirmed that children from age 4 understood the scale instructions.^4^

Next, subjects were given a practice trial to show them how to use the mouse to interact with the lineup faces. The practice trial was of a single South Asian female face. The RA explained that it was possible to click on and rotate the face if they wanted to, and subjects were given the opportunity to practice this movement. When the RA was satisfied that the subject understood how to interact with the face, they reminded the subject that they would next see some pictures of different men, and that James might be one of them, but he might not be any of them. Subjects were also told that they could use the mouse to explore the faces if they wanted to but did not have to. Subjects viewed a six-person lineup (either target-present or target-absent) and the RA asked: “Is one of the people James from the video or is James not one of the people here?” If the child chose to rotate and explore the faces during the lineup, our *Eyewitness Interactive Software* recorded, moment by moment, how the child rotated the lineup faces. Once subjects had stated whether James was one of the people present or was not present, subjects were asked how sure they were of their decision (i.e., gave a postidentification confidence judgment) using the water-cup rating scale. If subjects had identified someone in the lineup, they were told: “Remember, the more sure you are that is James, the more water will be in the cup.” If they said that James was not present, they were told: “The more sure you are that none of the pictures are James, the more water will be in the cup.” The scale was explained again in detail. Finally, the RA recorded the subjects’ responses, and recorded any technical problems when viewing the video or the lineup, or if the RA believed that the guardian had influenced the child’s answers. Children were offered a certificate as a reward for their participation.

### Results

We first examined average memory performance in the three age groups. Next, we examined memory reliability on the identification task by analyzing children’s explicit confidence judgements. We conducted CAC analysis^5^ and fit a signal-detection model to our data to examine whether children of different ages placed their confidence criteria in such a way as to maintain constant likelihood ratios. Finally, we explored an implicit measure of metacognition—the children’s viewing behavior during the lineup using the interactivity data—to examine whether children’s implicit expressions of certainty were informative of memory accuracy on a lineup task, as has typically shown to be true in the developmental literature using other decision-making tasks. In all analyses, the data were collapsed over the two sets of stimuli because we were interested in detecting effects that generalized over multiple encoding and test conditions.

#### Identification Responses

Table 1 shows the number of culprit identifications, filler identifications, and lineup rejections (“not present” responses) by subjects in young, middle, and late childhood at each confidence level in target-present and target-absent lineups. The overall correct ID rate of the culprit (displayed in the proportion row in Table 1) is equal to the total number of culprit IDs from target-present lineups divided by the total number of target-present lineups run in each age group. The number of innocent suspect IDs in target-absent lineups was estimated by dividing the number of target-absent filler IDs by the number of lineup members (i.e., six). That estimated value was then divided by the number of target-absent lineups to estimate the false ID rate in each age group. This estimation technique is a standard approach in the eyewitness literature and, when the target-absent lineup is fair, returns the same mean estimate of the number of innocent suspect identifications as predesignating a single individual to be the innocent suspect. The overall correct ID rates were .32, .43, and .55 for those in young, middle, and late childhood, respectively. The corresponding overall false ID rates were all .06 for those in young, middle, and late childhood. Thus, even without performing ROC analysis, it is clear that ability to discriminate between guilty and innocent suspects improved with age, and this was attributable to an increase in correct IDs with age.[Table tbl1]

It is important to note here that the witness literature has traditionally concluded that children aged from about 5 years are just as likely as their older peers (and even adults) to make a correct identification of a guilty suspect in a target-present lineup, and that age differences in lineup identifications are limited to older children making fewer mistaken identifications of innocent suspects from target-absent lineups (e.g., Dunlevy & Cherryman, 2013; Havard & Memon, 2013; Pozzulo & Lindsay, 1998). Those results may seem somewhat surprising to basic science researchers, given what is known in the developmental literature about the maturation of memory throughout childhood (e.g., Schneider & Ornstein, 2015). Indeed, more recent eyewitness child studies and a meta-analysis have found correct identifications of guilty suspects in target-present lineups increase with age (Brewer & Day, 2005; Fitzgerald et al., 2014; Fitzgerald & Price, 2015; Keast et al., 2007), and correct rejections of target-absent lineups increase slightly, but not significantly, with age (Fitzgerald & Price, 2015). The patterns that we observed in the ID responses replicate the more recent eyewitness findings.

#### ROC Analysis

**Figure 3 fig3:**
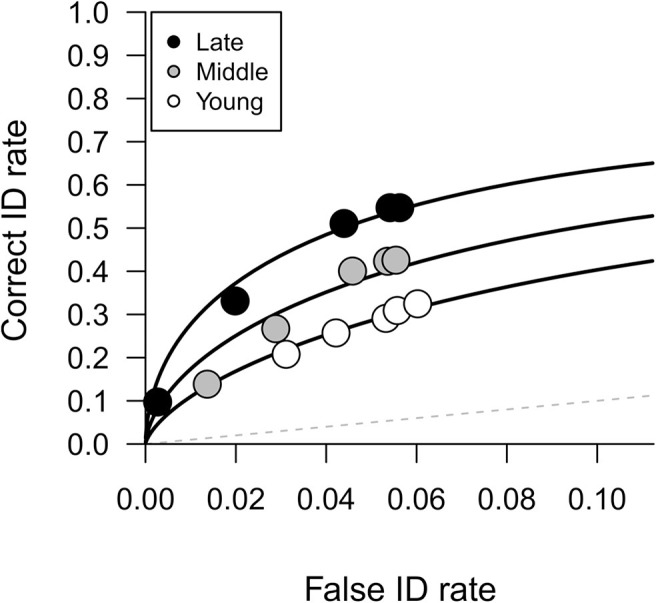
Young, Middle, and Late Childhood Receiver Operating Characte-ristic Data, Plotted Using Postidentification Confidence Judgments *Note.* The circles are the empirical data, and the lines of best fit were generated using the Independent Observations model fit to the data. The bottom *x* axis shows the estimated false ID rate of innocent suspects. The dashed line indicates chance-level performance.

We conducted ROC analysis to measure memory discrimination accuracy—participants’ collective ability to discriminate between guilty and innocent suspects. Figure 3 shows the ROC curves for subjects in young, middle, and late childhood (see Mickes et al., 2012, for a tutorial). Each ROC curve plots correct and false ID rates over decreasing levels of postidentification confidence, and confidence is used as a proxy for response bias. Partial area under the curve (pAUC) values were computed using a culprit-absent filler ID cutoff (i.e., specificity) of .67 with the statistical package pROC (Robin et al., 2011). The pAUC values were significantly larger for those in late childhood (pAUC = .12) than middle childhood (pAUC = .08, *D* = 3.09, *p* = .002) and young childhood (pAUC = .06, *D* = 5.54, *p* < .001). The pAUC values were also significantly larger for those in middle childhood than young childhood (*D* = 2.52, *p* = .012). Again, this demonstrates that memory discrimination accuracy improves through childhood.[Fig fig3]

#### Explicit Confidence Judgments

##### CAC Analysis

**Figure 4 fig4:**
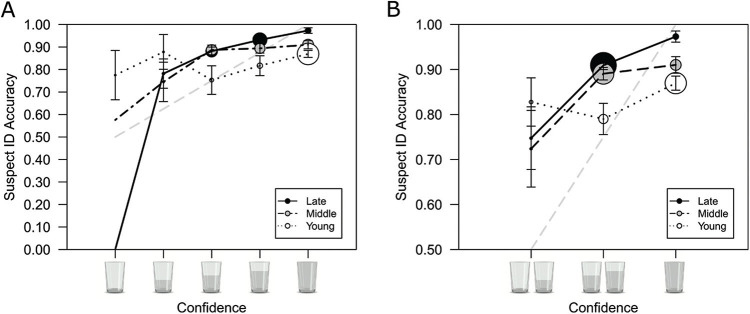
Young, Middle, and Late Childhood Confidence Accuracy Characteristic Data, Plotted Using Postidentification Confidence Judgments on (A) the 5-Point Cup Scale and (B) a Collapsed 3-Point Cup Scale *Note.* Bars represent standard errors, estimated using a bootstrap procedure (see Seale-Carlisle & Mickes, 2016). The dashed line indicates chance-level performance at the lowest confidence bin and perfect performance at the highest confidence bin. The size of the circles represents the number of suspect IDs at a given level of confidence, relative to the number of suspect IDs given at other levels of confidence (Seale-Carlisle, Wetmore, et al., 2019).

Next, we examined the reliability of children’s identification decisions by analyzing the relationship between confidence and accuracy, using CAC analysis. We plotted CAC curves for young, middle, and late childhood groups. First, we plotted CAC curves across the 5-point water cup rating scale (see Figure 4A). For each confidence level, we calculated suspect ID accuracy using the formula (correct ID rate)/(correct ID rate + ∼false ID rate), where ∼false ID rate refers to the estimated innocent suspect ID rate which is calculated by dividing filler IDs from target-absent lineups by the number of lineup members (6), and then dividing that by the number of target-absent lineups (Mickes, 2015). The CAC plot indicates that ability to assign appropriate confidence judgments that reflect likely suspect ID accuracy improved with age. In young childhood, there was no relationship between confidence and accuracy, but, qualitatively, in both middle and late childhood suspect ID accuracy increased with confidence. There were too few suspect IDs made with low and medium confidence to estimate standard error bars using a bootstrapping procedure. Therefore, we binned the data into low (empty cup, ¼ full cup), medium (½ full cup, ¾ full cup), and high (full cup) levels of confidence and calculated suspect ID accuracy for each bin (e.g., Mickes, 2015); as shown in Figure 4B. In late childhood, high-confidence IDs were more accurate than medium-confidence IDs, which were more accurate than the low-confidence IDs. In middle childhood, medium- and high-confidence IDs were more accurate than low-confidence IDs, although it is worth noting that the vast majority of suspect IDs for those in middle childhood were made with middle and high confidence. In young childhood, there was no difference in suspect ID accuracy at low, medium, or high confidence.[Fig fig4]

Considering high-confidence suspect IDs, these were more accurate in late (97%) than middle (91%), and in middle than young (87%) childhood. Nevertheless, all three age groups achieved high suspect ID accuracy at high confidence on this task. It is also clear from the size of the circles in Figure 4B that frequency of high-confidence suspect IDs decreased with age. In young childhood, there were many high-confidence suspect IDs, fewer in middle childhood, and fewest in late childhood. Considering low-confidence suspect IDs, in all three age groups, suspect ID accuracy was reasonable (83% accurate in young, 72% in middle, and 75% in late childhood). It is not uncommon for adult participants to make low-confidence judgments even though their objective suspect ID accuracy is above chance accuracy (i.e., above 50%; e.g., see Wixted & Wells, 2017, for a review). Here, our finding of reasonable performance at low confidence may be partly attributable to collapsing the data to form a 3-point scale. When accuracy is calculated for the lowest “empty cup” confidence rating on the 5-point scale, suspect ID accuracy is closer to chance (i.e., 50%) accuracy in the middle (58% accurate) and late (0% accurate) childhood groups but not the young childhood group (78% accurate).

Finally, we conducted a further (exploratory, not preregistered) analysis to explore whether the strong confidence–accuracy relationship in late childhood could be accounted for by the older children in this age group (see the Appendix). A CAC analysis of those in young-late (aged 10–12) and late-late (aged 13–17) childhood revealed no difference in the confidence–accuracy relationship in these two groups, indicating that the 10- to 12-year-olds, like the 13- to 17-year-olds, were already skilled at monitoring their memory and able to assign appropriate confidence judgments that reflected their suspect ID accuracy.

Overall, our CAC analysis replicates our findings from our reanalysis of Keast et al. (2007) and the six list-learning memory studies and indicates that confidence is informative of suspect ID accuracy in children from aged 10. In addition, our analysis indicates that there are improvements in memory-monitoring skills through childhood. Children from middle childhood (i.e., from around aged 8) are beginning to be able to make reliable suspect IDs because their confidence (low compared with medium and high) can be informative about likely accuracy. Next, we fit a theoretical signal-detection model to understand why the confidence–accuracy relationship improved from young to late childhood.

##### Constant Likelihood Ratio Signal-Detection Model

To examine how children of different ages place their confidence criteria, we fit a signal-detection model to the data in each of the three age groups (e.g., Wixted & Mickes, 2014). Recall that research has shown that adults “fan out” their confidence criteria across a memory strength continuum in conditions yielding poorer memory discriminability. Behaving in this way means that adults place their decision criteria optimally to maintain a constant likelihood of accuracy at each level of confidence over hard (poorer discrimination e.g., long viewing distance) and easy (better discrimination e.g., short viewing distance) conditions. Put another way, a constant likelihood ratio signal-detection model can account for such behavior in adults. Here, we tested whether those in young and middle childhood “fan out” their confidence criteria to account for their poorer discrimination accuracy compared with the late childhood group, and if those in young childhood “fan out” their confidence criteria to account for their poorer discrimination accuracy compared with the and middle childhood group. In this section, we explain the basic signal-detection model fit, examine how the three age groups place their decision criteria by inspecting the model-generated parameters and likelihood ratios, and then fit the model constraining the confidence criteria across age groups to achieve constant likelihood ratios to statistically test whether children behave in a way predicted by the constant likelihood ratio model.

The model uses counts of culprit, filler, and reject identification decisions made at different levels of postidentification confidence in target-present and target-absent lineups to estimate parameters: discriminability (i.e., ability to discriminate between faces that have and have not been seen before) and a set of confidence criteria (*c_1_* − *c_3_*). The model assumes that when a witness views the faces in a lineup, each face has some memory strength value. In fair lineups, like ours, these memory strength values can be represented by two Gaussian distributions: one for guilty suspects (µ*_guilty_*), and one for innocent suspects and fillers (µ*_innocent_*). µ*_guilty_* lies higher on the memory strength axis than µ*_innocent_* because, on average, guilty suspects are associated with a greater memory strength than innocent suspects and fillers who have not been seen before. Memory discriminability is measured by the distance between the two distributions (*d'*), with less overlap indicating better discriminability. Notably, for this analysis, the model conceptualizes the confidence ratings provided by witnesses as different decision criteria. We used the same confidence bins as in the 3-point scale CAC analysis (*c_1_*: low confidence, *c_2_*: medium confidence, and *c_3_*: high confidence). The model assumes that the witness picks the face with the strongest memory signal, and if no face has a memory strength value that exceeds the lowest decision criterion (*c_1_*), the witness states “Not Present.” This is known as the independent observations rule (Colloff et al., 2018; Wixted et al., 2018).

We fit an equal-variance model and set the variances for the innocent and guilty distributions to 1. Although the variances of the distributions typically differ in practice, when the variances are unequal and Gaussian, the likelihood ratio model does not make simple predictions about the optimal placement of the decision criteria across conditions that differ in *d'*. When an unequal variance model is used, there are multiple locations on the memory strength axis that return the same likelihood ratios. As such, the idea that people behave in a way to maintain constant likelihood ratios on an unequal variance model seems implausible (Stretch & Wixted, 1998). Here, we took the usual approach and fit an equal variance model, so that the predictions of the likelihood ratio model are unambiguous (e.g., Semmler et al., 2018; Stretch & Wixted, 1998).

**Figure 5 fig5:**
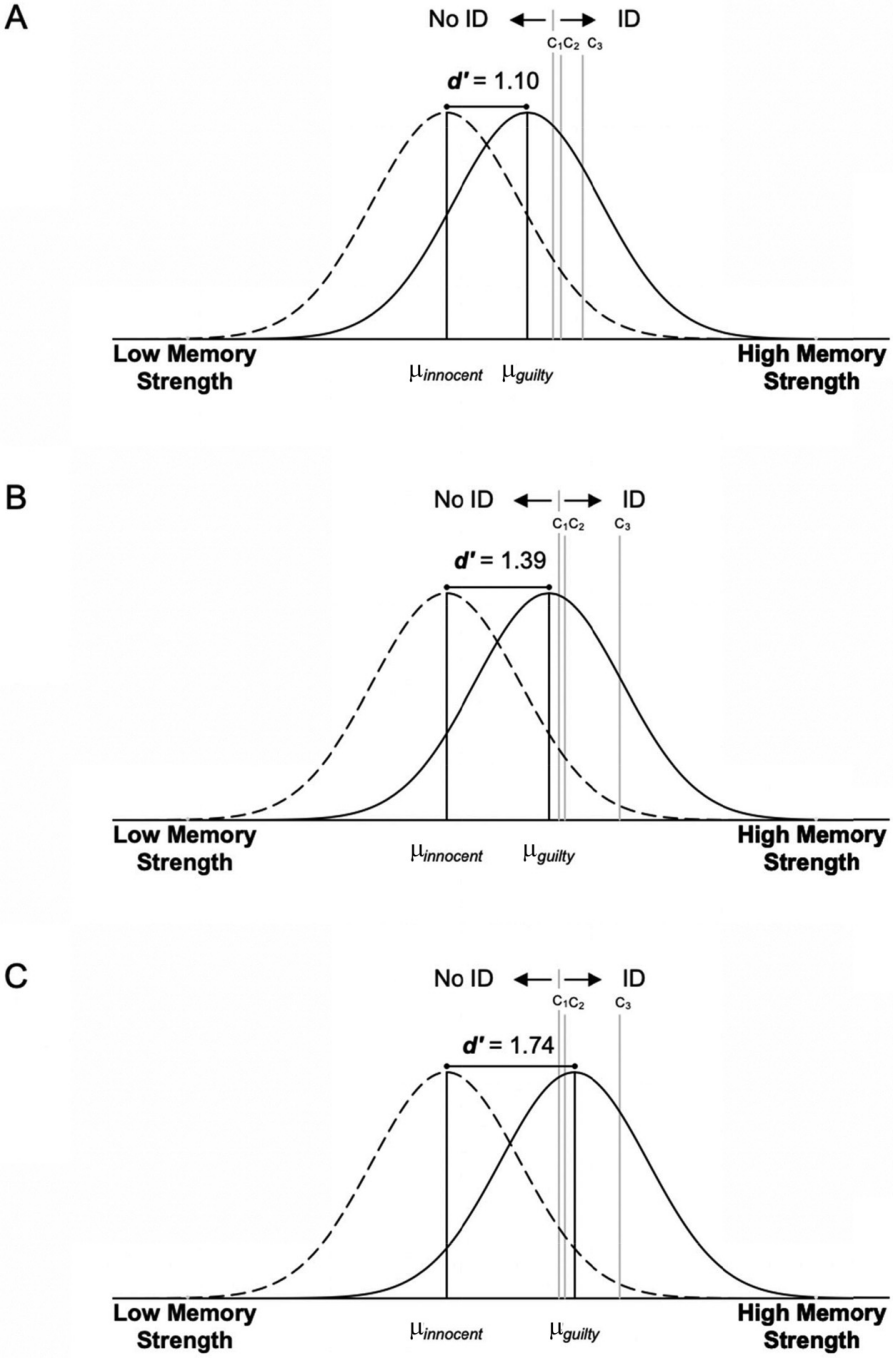
Innocent and Guilty Distributions and Confidence Criteria (c_1_, c_2_, c_3_) for Children in (A) Young, (B) Middle, and (C) Late Childhood Using the Best-Fitting Equal Variance Signal-Detection Model Parameters

Figure 5 displays the best-fitting model estimated parameters for each age group. The model predicted values differed significantly from the observed values for the middle and late childhood data (young: χ^2^[5] = 2.69, *p* = .75; middle: χ^2^[5] = 17.04, *p* = .004; late: χ^2^[5] = 15.44, *p* = .009). One possibility is that the data would be better explained by an unequal-variance model in which the variance of the target distribution is smaller than the lure distribution. Although allowing for unequal-variance did significantly improve the fit for the middle childhood group, χ^2^(1) = 7.27, *p* = .007, it did not for the late childhood group, χ^2^(1) = 2.48, *p* = .12, and in both groups the unequal variance model predicted values still differed significantly from the observed values (middle: χ^2^[4] = 9.77, *p* = .04; late: χ^2^[4] = 12.96, *p* = .01). We examined where the equal-variance model predictions most deviated from the observed ID frequencies. For both the middle and late childhood groups the model underestimated culprit IDs and overestimated target-present filler IDs at the medium confidence level (½ full cup and ¾ full cup). It is not clear why children seemed to prefer using the middle of the confidence scale when making culprit IDs, but that trend is also evident on Figure 4B, as illustrated by the larger size of the medium- compared with the low- and high-confidence points for the middle and late childhood groups. Nevertheless, whatever the reason for the poorer fit, the model adequately captured the trends in the data in all three age groups; the model-predicted lines of best fit drawn on Figure 3 closely follow the empirical data points and so the model is deemed to be appropriate to interpret the results.[Fig fig5]

Looking at Figure 5, the overlap in the guilty and innocent distributions clearly decreases (i.e., *d'* increases) with age, indicating an improvement in memory discrimination accuracy. But how does the location of the confidence criteria differ in the three age groups?^6^ Those in young and middle childhood did not place their decision criterion optimally in accordance with a constant likelihood ratio model. If those in young and middle childhood were placing their criteria optimally to account for their poorer discriminability, they would fan out their decision criterion along the memory axis compared with those in late childhood, placing their most conservative decision criteria (*c_3_*) at a more conservative location and their liberal decision criteria (*c_1_*) at a more liberal location. Instead, those in young and middle childhood groups set each of their criterion in increasingly liberal positions compared with those in late childhood. Those in young childhood also set each of their criterion in increasingly liberal positions compared with those in middle childhood. This is particularly evident for the high-confidence criterion (*c_3_*), which young children place in a much more liberal position (i.e., leftward on the memory axis), compared with those in middle and late childhood.

Nonoptimal placement of the decision criterion by children in young and middle childhood, as elucidated by the model-estimated parameters, explains why the CAC curve for those in young childhood is flat, and why the CAC curve for those in middle childhood is not as steep as the CAC curve for those in late childhood (Figure 4B). Those in young childhood made high-confidence suspect IDs when the likelihood ratio of correct (guilty) to incorrect (innocent or filler) IDs was low, as indicated by the large areas of the guilty and innocent distributions that fall above *c_3_* (Figure 5A). They made low-confidence suspect IDs when the likelihood was also low. The likelihood ratios associated with *c_1_*, *c_2_* and *c_3_* for the young childhood group were estimated to be 2.68, 3.01, and 4.18, respectively. Conversely, those in late childhood made high-confidence suspect IDs when the likelihood ratio of correct to incorrect IDs was high, as indicated by the large area of the guilty distribution, but the small area of the innocent distribution, that exceeds *c_3_* (Figure 5C). They also made low confidence IDs when the likelihood ratio was low. The likelihood ratios associated with *c_1_*, *c_2_*, and *c_3_* for the late childhood group were estimated to be 3.40, 4.24, and 36.48, respectively. Those in middle childhood group performed between those two extremes: they made high-confidence suspect IDs when the likelihood ratio of correct to incorrect IDs was moderately high, as indicated by the moderately large area of the guilty distribution, but the small area of the innocent distribution, that exceeds *c_3_* (Figure 5B). They also made low confidence IDs when the likelihood ratio was low. The likelihood ratios associated with *c_1_*, *c_2_* and *c_3_* for the middle childhood group were estimated to be 3.14, 3.62, and 10.08, respectively. Clearly, the likelihood ratios increase, indicating a higher likelihood ratio with higher confidence, but compared with those in late childhood, those in middle childhood made high confidence judgments when the likelihood ratio of correct to incorrect IDs was not as high.

To statistically test the observation that the pattern of data across age groups is inconsistent with a constant likelihood ratio model, we constrained the confidence criteria across two age groups so that they had the same likelihood ratios. For example, we estimated *c_1_*, *c_2_*, and *c_3_* in the late childhood group and then constrained that *c_1_*, *c_2_*, and *c_3_* in the middle childhood group be placed in positions on the memory strength axis to maintain the same likelihood ratios as in the late childhood group. Constraining the confidence criteria markedly and significantly worsened the fit for all three pairwise comparisons (late vs. middle: χ^2^[3] = 51.09, *p* < .001; late vs. young: χ^2^[3] = 238.05, *p* < .001; middle vs. young: χ^2^[3] = 77.53, *p* < .001). This indicates that the young and middle childhood groups did not place their decision-criterion in accordance with a constant likelihood ratio model to account for their poorer memory performance.

Considered together, the CAC analysis and model-fitting show that the ability to assign appropriate confidence judgments is better in middle compared with young childhood, and despite emerging metacognitive abilities, those in middle childhood are still slightly less accurate at high levels of confidence, compared with those in late childhood. This appears to be because—inconsistent with a constant likelihood ratio account—those in middle childhood, but especially those in young childhood, place their decision criterion more liberally than is necessary to achieve the same level of accuracy at each level of confidence as the late childhood group.

Those in young childhood did not show a meaningful relationship between confidence and accuracy. But were younger children able to appropriately express uncertainty implicitly, such as via their viewing behavior during the interactive lineup? We examined the children’s interactivity data next.

#### Interactivity as an Implicit Measure of Metacognition

Recall that basic developmental research has found that young children from age 3 can appropriately express uncertainty *implicitly* without full awareness, using gestures like shaking their head, shrugging their shoulders (Kim et al., 2016). Implicit measures of metacognition on eyewitness identification tasks with children have seldom been considered. In the adult eyewitness literature, IDs are more likely to be accurate when cognitive processes are automatic (e.g., the face “stood out”) and fast, whereas IDs are more likely to be inaccurate when cognitive processes are considered (e.g., process of elimination decisions) and slow (Dunning & Stern, 1994). A number of experiments have found that faster decisions in adults yield more accurate suspect IDs (e.g., Sauer et al., 2008; Sauerland & Sporer, 2009; Seale-Carlisle, Colloff, et al., 2019), a finding that has been replicated with children from age 4 (Bruer & Pozzulo, 2014) and from age 8 (Brewer & Day, 2005).

We explored whether the way in which children interacted with the lineup faces changed with age, or whether viewing behavior (signaling automatic, fast processing) could be informative about ID accuracy. Specifically, we examined whether children’s suspect IDs were more accurate when they first clicked on and rotated the suspect instead a filler, and whether discrimination accuracy and suspect ID accuracy was better for fast (less time spent interacting) than slow (more time spent interacting) IDs.

First, we conducted a preliminary analysis. The proportion of children who interacted with at least one face differed in young (62%), middle (75%), and late childhood (76%), *F*(2, 2202) = 21.14, *p* < .001, $\eta_{p}^{2}$ = .019. Those in young childhood were significantly less likely to interact than those in middle, *t*(1447.5) = 5.57, *p* < .001, *d* = .29, and late, *t*(1354.9) = 5.47, *p* < .001, *d* = .30, childhood. Those in middle and late childhood were similarly likely to interact, *t*(1381.3) = .24, *p* = .809, *d* = .01. We examined the viewing behavior of subjects who did interact (*n* = 1,569).

To explore suspect ID accuracy for seemingly automatic decisions in which the face stood out to the participant, we examined whether the first face that children rotated could differentiate between correct IDs of culprits and false IDs of innocent suspects. When children clicked on a face and rotated it, all of the faces in the lineup moved together. Children were not told that all of the faces would move together, so our measure here reflects children’s interest in a given face that they chose to click on and rotate first. We estimated the number of children who first rotated an innocent suspect in target-absent lineups by dividing the number of children who interacted with any face in the target-absent lineups by the number of lineup members (six). Similarly, we estimated the number of children who first rotated a filler face in target-absent lineups by dividing the number of children who interacted with any face in the target-absent lineups by the number of lineup members (six), and then multiplying by the number of lineup members who were not the innocent suspect (five).^7^ Interestingly, in all three age groups, the first face that children rotated was informative about suspect ID accuracy. Three 2 (suspect ID: correct, false) × 2 (interact first: suspect, filler) two-way chi-square analyses indicated that those in young childhood who made a correct ID of the culprit were 3.76 times more likely to have interacted with the suspect first instead of a filler, than those who made a false ID of an innocent suspect, χ^2^(1, *N* = 159) = 12.82, *p* < .001, odds ratio (*OR*) 3.76, 95% CI [1.70, 8.83]. In middle childhood, those who made a correct ID of the culprit were 4.24 times more likely to have interacted with the suspect first, than those who made a false ID of an innocent suspect, χ^2^(1, *N* = 255) = 25.19, *p* < .001, *OR* = 4.24, 95% CI [2.30, 8.11]. Similarly, in late childhood, those who made a correct ID of the culprit were 4.98 times more likely to have interacted with the suspect first, than those who made a false ID of an innocent suspect, χ^2^(1, *N* = 238) = 27.55, *p* < .001, *OR* = 4.98, 95% CI [2.58, 10.05]. As such, if a child first interacted with the suspect, then this is an indicator of likely suspect ID accuracy; namely, that the suspect is the real culprit.

Next, we were interested in exploring suspect ID accuracy for seemingly automatic fast decisions compared with considered slow decisions. In each age group, we examined the relationship between the amount of interaction and suspect ID accuracy, and the amount of interaction and memory discrimination accuracy—ability to discriminate between innocent and guilty suspects, *d'*. We measured the overall length of time participants spent rotating the faces and, in each age group, created two interaction groups: high and low interaction, using a median split. For those in young childhood, the overall mean interaction time for low interactors was 21.54 s and for high interactors was 78.47 s. The overall mean interaction times for low- and high-interactors in middle childhood, and low- and high-interactors in late childhood were 16.50 and 66.03, and 14.36 and 59.14 s, respectively. The overall correct ID rate (of guilty suspects in target-present lineups) and false ID rates (of innocent suspects in target-absent lineups), suspect ID accuracy, and *d'* values for high and low interactors in each age group are displayed in Table 2. Suspect ID accuracy was higher for low compared with high interactors in each age group. Moreover, in each age group, there was a trend for low interactors to have better memory discrimination accuracy than high interactors, but the difference were not statistically significant when we computed the *G* statistic for the young (*G* = .71, *p* = .48), middle (*G* = .66, *p* = .51), or late (*G* = 1.58, *p* = .11) childhood groups (two-tailed, Gourevitch & Galanter, 1967; see also Mickes et al., 2014). More research is required with larger sample sizes in low and high interactor groups, but this provides preliminary evidence that something as simple as the amount of time taken exploring lineup faces, might be informative about the likely accuracy of witness identifications, even in young children.[Table tbl2]

## Discussion

We investigated the informativeness of children’s expressions of certainty, to better understand the apparent divide between basic and applied research. Our work illustrates that the divide between the literatures is more apparent than real. We conducted a reanalysis of the confidence–accuracy relationship in seven recognition memory studies in the basic and applied literatures, and further investigated the reliability of eyewitness identifications made by children in young (aged 4–6), middle (aged 7–9), and late (aged 10–17) childhood, by examining the relationship between confidence and accuracy, and by exploring whether viewing behavior during an interactive lineup was associated with suspect ID accuracy. Contrary to what is believed to be true in legal systems around the world, our reanalysis of the basic literature highlighted a strong confidence–accuracy relationship in children from aged 8 (and perhaps even from age 5). Our reanalysis of the eyewitness literature, and our own experiment highlights children’s confidence judgments were informative about accuracy on a lineup identification task in late childhood (from age 10), somewhat informative in middle childhood (from age 7), and some patterns of viewing behavior were associated with accuracy in all age groups (from age 4). Ability to discriminate guilty from innocent suspects improved with age.

### Metacognitive Development

Our findings have important implications for understanding children’s metacognitive development. Heretofore, the results of applied witness research were at odds with the developmental literature. The previous analytic methods used in the witness literature led researchers to conclude memory-monitoring skills for complex witnessed events emerged at a markedly later age than developmental research has found. We found that children’s certainty expressions can indicate likely accuracy, even on a complex task on which children have encoded an event and are later asked to identify a previously seen culprit from an identification parade. This suggests that the fundamental architecture of metacognition that has previously been evidenced in the developmental literature on relatively simple tasks (e.g., Destan et al., 2014; Hembacher & Ghetti, 2014; Shing et al., 2009) also underlies performance on complex memory tasks.

Yet, our results with young children indicate that proficiency to monitor accuracy is dependent on how certainty is measured. Young children in our experiment were unable to use confidence judgments (an explicit judgment) to reflect their likely suspect ID accuracy but did express certainty using their interactive behavior (implicit metacognition) during the lineup and those behaviors were associated with suspect ID accuracy. Developmental research has also evidenced implicit metacognitive monitoring in children aged 3 upward, who shrug (Kim et al., 2016) or ask for help when they are unsure (Ghetti et al., 2013). It appears that young children may be able to monitor the likely accuracy of their memories (Goupil et al., 2016; Monosov & Hikosaka, 2013) but have difficulty reliably transforming subjective confidence into a probabilistic scale (Ferrell & McGoey, 1980). Together, our exploratory analyses on children’s interactivity behavior, and the previous developmental literature (e.g., Ghetti et al., 2013; Kim et al., 2016), indicate that measuring implicit metacognition could revolutionize the way in which researchers (both basic and applied) and legal practitioners (e.g., police officers, jurors) assess the certainty and accuracy of memories in young children. From a signal-detection memory perspective, measures of certainty can be considered to be proxies for memory strength, with explicit judgments (e.g., confidence) requiring awareness of certainty or the strength of the memory signal, and implicit measures (e.g., interactivity) not requiring (full) awareness (e.g., Kim et al., 2016). There are many other measures of implicit metacognition that could be reliable proxies for memory strength (e.g., response times, pupil dilations, grip strength). Future research should examine which measures of implicit metacognition are most predictive of accuracy in children of different ages.

The correspondence between confidence and accuracy is seldom measured in basic research but can convey important information about memory monitoring skills (Juslin et al., 1996). The correspondence between confidence and accuracy has been measured in applied research, but eyewitness research has traditionally relied on statistical techniques that can underestimate the relationship between confidence and accuracy (for a review, see Wixted & Wells, 2017). Across basic and applied literatures there was a relationship between confidence and accuracy in children, from at least age 8, because confidence increased monotonically with accuracy. In the basic literature, children from age 5 were generally overconfident at high confidence (e.g., around 85% accurate, when they were highly confident), but their expression of confidence still provided considerable information about the likely accuracy of their recognition memory decision. In our eyewitness experiment, there was no relationship between confidence and accuracy in young children (aged around 5 years old), but we gave children relatively brief instructions on how to use the confidence scale and the instruction for children who said the culprit was “Not Present” was somewhat complex (i.e., a double negation). As such, it is possible that given more detailed or simplistic instruction and practice trials (e.g., Hembacher & Ghetti, 2014); confidence judgments made by child witnesses aged 5 could be *more* informative about their likely accuracy than we found here.

### Practical and Theoretical Implications

Why is this important? These findings are important because they unify the basic and applied literature which have previously been at odds and have significant practical and theoretical implications. Practically, our data show that children from around age 7 could be reliable eyewitnesses, because their confidence (low compared with medium and high) is related to suspect ID accuracy and suspect IDs made with high confidence are likely to be accurate. Our data and the data from Keast et al. further show that children from age 10 are reliable and display a strong confidence–accuracy relationship. These findings portray a strikingly more positive picture of child witness reliability than the previous witness literature and reconcile findings with the basic literature. Memory evidence from children is often deemed by legal decision-makers to be unreliable and is disproportionality less likely to be believed than memory evidence from adults (e.g., Kassin et al., 2001; Knutsson & Allwood, 2014; Newcombe & Bransgrove, 2007). Thus, the accuracy of child memory evidence might currently be underestimated in the legal system. Notably, in our experiment, in middle (from age 7) and late childhood (from age 10) groups, confidence judgments provided more information about likely suspect ID accuracy than age alone. Knowing that a confident child witness is more likely to be accurate than a less confident witness provides important information for legal decision-makers about how to proceed with their inquiry or how much trust to place in identification evidence (Brewer & Wells, 2006).

Theoretically, our work to unify the literatures can help to advance mechanistic understanding of memory monitoring. In adults, a constant likelihood ratio signal-detection model has been proposed to explain the meaningful relationship between confidence and recognition memory accuracy, even in conditions of poorer memory discrimination accuracy (Colloff et al., 2017; Semmler et al., 2018; Stretch & Wixted, 1998). This account suggests that adults fan out their confidence criteria across a memory strength continuum in conditions yielding poorer memory discriminability, such that accuracy for a given level of confidence remains the same over hard (poorer discrimination) and easy (better discrimination) conditions. Children in young and middle childhood did not fan out their confidence criteria in a way predicted by a constant likelihood ratio model, to account for their poorer memory discriminability. This can explain why, in the CAC analysis, those in middle childhood were slightly less accurate than those in late childhood at the highest levels of confidence; At the highest level of confidence, those in middle childhood placed their high-confidence criteria (*c_3_*) in a more liberal position than was necessary to achieve a higher likelihood ratio of correct to incorrect IDs. It can also explain the lack of relationship between confidence and accuracy in young children, because those in young childhood, placed their decision criterion in such a way that there was a relatively similar likelihood ratio of correct to incorrect IDs at each confidence level.^8^

One theory suggests that adults learn how to place their confidence criteria optimally through a lifetime of error feedback training about the circumstances in which their memories are and are not accurate (Mickes et al., 2011; Stretch & Wixted, 1998; see also Lindsay et al., 1998). Indeed, developmental observational studies indicate that direct instruction, such as teachers providing metacognitive strategies, is associated with improved metacognitive monitoring on memory tasks in children (Coffman et al., 2008; also see Roebers, 2017). Future research should further test the predictions of the constant likelihood ratio signal-detection model by testing children under both hard (poorer discrimination) and easy (better discrimination) conditions. Moreover, future research could test the causal role of feedback on metacognitive performance in children of different ages to develop a unified theory that can precisely explain the error feedback process by which metacognitive skills improve throughout development.

Although these findings have significant applied and theoretical implications, it is important to remember that the confidence–accuracy relationship is likely to be influenced by underlying memory performance (i.e., *d'*). For example, a meaningful confidence–accuracy relationship is unlikely when memory performance becomes very poor, or is around chance levels (e.g., Fleming & Lau, 2014; Kruger & Dunning, 1999; Sauer et al., 2019). In the basic metacognitive literature, researchers are beginning to separately measure two elements of metacognitive performance in adults—*sensitivity* and *efficiency* (Maniscalco & Lau, 2012). Sensitivity is the ability to distinguish between one’s own correct and incorrect responses with certainty judgments and can be measured by *meta-d'.* Efficiency accounts for the influence of memory ability (*d'*) on metacognitive sensitivity by computing a numerical comparison between the two (*meta-d'/d'*). The current literature cannot yet tell us about the development of metacognitive *efficiency* in children from age 4 to 17 on complex memory tasks. For instance, when viewing the flat CAC curve for young children, metacognitive performance appears poor. Yet, metacognitive performance might be better than it appears, when performance on the underlying memory task is taken into account and metacognitive efficiency is calculated. Ongoing work in our lab is beginning to address that possibility. Nevertheless, from an applied perspective, a younger child eyewitness *is* likely to have poorer memory discrimination accuracy than those in middle and late childhood and, as such, the CAC analysis provides vital information that legal decisions ought to know: Namely, the likely accuracy of an identification made with a particular level of confidence.

Moreover, our interactivity findings are useful to advance theory about how people make recognition memory decisions. Children in all age groups were more likely to make accurate suspect IDs when they interacted first with the suspect’s face instead of the other filler faces. As such, interactivity behavior might provide more information about what people remember than the overt recognition decision (yes/no) alone (for similar ideas, see Bruer et al., 2017; Hannula et al., 2012). We also found a trend that subjects were better able to tell the difference between innocent and guilty suspects, when they spent *less* time interacting. In other related work using interactive lineups, we have manipulated the encoding angle of a culprit and found that adults had better memory discrimination accuracy when they spent a *longer* proportion of time rotating the lineup faces to view the side of the culprit’s face that they had viewed at encoding (Colloff et al., 2020). Moreover, interactive lineups can substantially improve adult memory discrimination accuracy compared with traditional lineups which are composed of static photos of the lineup members facing the camera (Colloff et al., in press). Together, it seems that interacting for a longer length of time did not necessarily harm memory discrimination accuracy in our sample of children. Rather, witnesses with strong and weak memories might interact in different ways. It is possible that witnesses with strong memories might interact for disconfirming feedback, to check whether their best candidate in the lineup sufficiently matches their memory, which is a relatively quick process. Conversely, those with weak memories might be exploring whether any particular angle or further examination of the faces might provide a stronger match to memory, which is a relatively slow process. This explanation is concordant with the existing literature that indicates that correct identifications, made by people with strong memories, are likely to be made quickly (e.g., Dobolyi & Dodson, 2018; Sauerland & Sporer, 2009; Seale-Carlisle, Colloff, et al., 2019). This post hoc explanation of our results should be tested in future research. Greater theoretical understanding of how witnesses make decisions from lineups is of paramount importance because theory should be used to advance appropriate procedures to improve identification accuracy (Gronlund et al., 2015; Wixted & Mickes, 2014).

### Unifying the Basic and Applied Literatures

Improving theoretical understanding of memory requires input from both basic and applied researchers. Without communication between fields, progress is stymied (Albright & Rakoff, 2020; Mickes & Wixted, in press). Here, we have shown how unification of literatures (memory, developmental, metacognition) is necessary to answer the important applied question of how to determine the reliability of children’s lineup identification decisions and how techniques from the applied literature can be used to further our understanding about memory monitoring. With greater communication and better integrated research approaches across fields, inconsistent findings could have been resolved more quickly, and basic science findings that have been limited to laboratory settings could have already been extended to have impact in applied settings (for similar ideas see also Gronlund & Benjamin, 2018; Lane & Meissner, 2008).

The key take-home message is that the longstanding contradiction between the basic and applied literatures does not appear to be real. Contrary to the conclusions of previous witness literature and established beliefs in the criminal justice system, it seems that suspect identifications made by children can be reliable when appropriate metacognitive measures (informed by the developmental and metacognitive literatures) are used to assess accuracy. At least from age 7, a child’s explicit expression of confidence provides information about the likely accuracy of a suspect ID. As others have put it: “eyewitness memory confidence is a useful but imperfect indicator of the truth” (p. 113, Roediger et al., 2012; see also Brewer & Wells, 2006). Further investigation of measures of implicit metacognition should prove fruitful in determining the accuracy of recognition memory decisions made by younger children. It is imperative that contradictory findings in basic and applied literatures are reconciled to advance theoretical understanding. Similarly, given what is at stake—the wrongful conviction of innocent people, or guilty people being free to commit further crimes—it is imperative that we continue to use evidence from basic and applied science to inform and investigate novel ways to determine the likely accuracy of child memory evidence.

## Figures and Tables

**Table 1 tbl1:** Identification Response Frequencies Made by Subjects in Young, Middle, and Late Childhood at Different Postidentification Confidence Levels in Target-Present and Target-Absent Lineups

	Young					Middle					Late				
	Target present			Target absent		Target present			Target absent		Target present			Target absent	
Confidence	Culprit	Filler	Reject	Filler	Reject	Culprit	Filler	Reject	Filler	Reject	Culprit	Filler	Reject	Filler	Reject
Empty cup	6	9	32	9	36	1	3	6	5	11	0	1	0	4	3
¼ full cup	7	6	14	5	18	9	7	19	21	23	12	4	11	19	12
½ full cup	13	17	24	22	25	53	25	28	46	71	59	14	48	45	70
¾ full cup	19	15	23	22	21	51	25	50	41	84	77	10	35	32	76
Full cup	80	50	70	62	112	55	11	54	37	112	32	4	22	5	45
Total	125	97	163	120	212	169	71	157	150	301	180	33	116	105	206
Proportion	.32	.25	.42	.36	.64	.43	.18	.40	.33	.67	.55	.10	.35	.34	.66
*Note*. Confidence was collected using a pictorial 5-point water cup rating scale. Empty cup = “not at all sure” to Full cup = “very sure.” Because of rounding, proportions do not always appear to add up to 1.															

**Table 2 tbl2:** Correct and False Identification (ID) Rates and d', Along With 95% Confidence Intervals, in Low and High Interactors, as a Function of Age Group

Age group	Low interactors				High interactors			
	Correct ID rate	False ID rate	Suspect ID accuracy	*d*′ [95% CI]	Correct ID rate	False ID rate	Suspect ID accuracy	*d*′ [95% CI]
Young	.36	.06	.86	1.18 [0.72, 1.65]	.30	.07	.81	0.95 [0.51, 1.40]
Middle	.48	.06	.89	1.50 [1.14, 1.86]	.41	.06	.87	1.33 [0.96, 1.69]
Late	.65	.07	.91	1.89 [1.46, 2.31]	.46	.07	.87	1.38 [0.96, 1.80]

**Table A1 tbl3:** Identification Response Frequencies Made by Subjects in Young-Late (10–12) and Late-Late (13–17) Conditions at Different Postidentification Confidence Levels in Target-Present and Target-Absent Lineups

	Young-Late					Late-Late				
	Target present			Target absent		Target present			Target absent	
Confidence	Culprit	Filler	Reject	Filler	Reject	Culprit	Filler	Reject	Filler	Reject
Empty cup	0	1	0	1	3	0	0	2	3	0
¼ full cup	6	4	9	11	8	6	0	13	8	4
½ full cup	47	10	35	35	54	12	4	7	10	16
¾ full cup	59	7	28	26	63	18	3	1	6	13
Full cup	18	2	21	3	35	14	2	9	2	10
Total	130	24	93	76	163	50	9	32	29	43
Proportion	0.52	0.10	0.38	0.32	0.68	0.61	0.10	0.39	0.40	0.60
*Note*. Confidence was collected using a pictorial 5-point water cup rating scale. Empty cup = “not at all sure” to Full cup = “very sure.” When an equal-variance signal detection model was fit to these data, *d*′ was estimated to also be the same across the young-late and late-late groups.															

**Figure A1 fig6:**
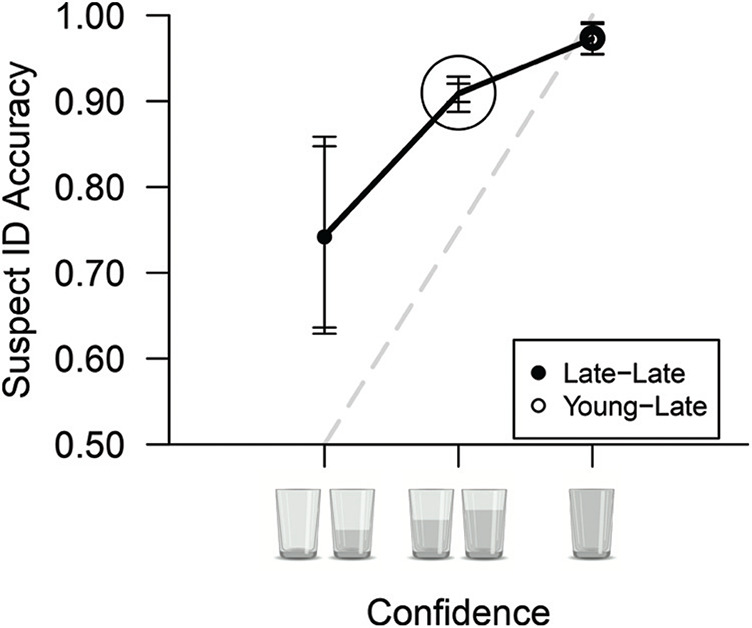
Young-Late and Late-Late Childhood Confidence Accuracy Characteristic Data Plotted Using Postidentification Confidence Judgments *Note.* Bars represent standard errors, estimated using a bootstrap procedure (see Seale-Carlisle & Mickes, 2016). The dashed line indicates chance-level performance at the lowest confidence bin and perfect performance at the highest confidence bin. The size of the circles represents the number of suspect IDs at a given level of confidence, relative to the number of suspect IDs given at other levels of confidence (Seale-Carlisle, Wetmore, et al., 2019).

## Footnotes

1 Note that, in the basic science literature, there is ongoing consideration of whether metacognition is a general trait-like ability, because metacognitive performance may or may not be consistent over time and different tasks or measures (e.g., Mazancieux et al., 2020; Steiner et al., 2020).

2 Note that using confidence as a proxy for response bias (or memory strength) is consistent with a signal-detection interpretation of memory. However, in the metacognitive literature, confidence judgments are often considered to be based on multiple extraneous factors, such as fluency or expertise.

3 The modal description for the male culprit tidying up children’s toys was: *male, White/Caucasian, aged 18–34, average/heavy build, medium eye color, dark hair color, short hair length, no facial hair, light/medium complexion, no distinctive features*. The modal description for the male culprit returning home with shopping and eating chocolate was: *male, White/Caucasian, aged 18–34, average build, medium eye color, medium hair color, short hair length, no/some facial hair, light complexion, no distinctive features*.

4 The children’s preidentification confidence ratings will not be discussed further, because they are beyond the scope of the current study; these data are included in our open-access datafile (https://osf.io/3zjd6/).

5 As per our preregistered plan, we also calculated calibration statistics that have typically been calculated in the witness literature and those analyses are available on the open science framework (https://osf.io/3zjd6/).

6 Because the *c_1_* and *c_2_* criteria are so closely placed at all ages (Figure 5), it would also be justifiable to fit a model using just two confidence criteria. When we conducted the analysis using a model with two confidence criteria (*c_1_*: empty cup, ¼ cup, ½ cup, ¾ cup; and *c_2_*: full cup), the results were that same as when we conducted the analysis using the model with 3 confidence criteria.

7 Another way to conduct this analysis is to assume that the culprit’s replacement in the target-absent lineup is the innocent suspect. If we use this method, the pattern of the results is the same.

8 Note that the chi-square goodness-of-fit values indicated that the observed data departed from the predictions of the simple equal-variance signal-detection model to a marginally significant degree in the middle and late childhood groups. This suggests that a more complex model might fit the data better and might be more appropriate to interpret these data.

## Exploratory CAC Analysis for Young-Late and Late-Late Childhood Groups

We plotted additional confidence accuracy characteristic (CAC) curves for young-late (aged 10–12, *M*_age_ = 10.73, *SD* = .76), and late-late (aged 13–17, *M*_age_ = 13.92, *SD* = 1.16) childhood groups (see Figure A1). We constructed the curves using the same method that we used for the preregistered CAC analysis that we report in the main results section. The number of suspect identifications, filler identifications, and lineup rejections (“not present” responses) by subjects in the young-late and late-late childhood groups at each confidence level in culprit-present and culprit-absent lineups are shown in Table A1. The CAC plot shows that the curves for young-late and late-late overlap at every level of confidence. This indicates that the 10- to 12-year-olds, like the 13- to 17-year-olds, already had the metacognitive awareness to assign appropriate confidence judgments that reflect their suspect ID accuracy.[Fig fig6][Table tbl3]
